# Supplementary material for: Greater Greenland Ice Sheet contribution to global sea level rise in CMIP6
Source: Nat Commun. 2020 Dec 15;11:6289. doi: 10.1038/s41467-020-20011-8 (PMC7738669; doi:10.1038/s41467-020-20011-8)
Supplement: Supplementary file 1 — Supplementary Information [file 41467_2020_20011_MOESM1_ESM.pdf]

Supplementary Material - Greater Greenland Ice Sheet contribution to global sea level rise in CMIP6

Stefan Hofer,[1,2*] Charlotte Lang,[2] Charles Amory,[2] Christoph Kittel,[2] Alison Delhasse,[2] Andrew Tedstone,[3] and Xavier Fettweis[2]

[1]Department of Geosciences, University of Oslo, Oslo, Norway
[2]SPHERES research units, Geography Department, University of Liège, Belgium
[3]Department of Geosciences, University of Fribourg, Switzerland

[*]Corresponding author: Stefan Hofer, stefan.hofer@geo.uio.no

Supplementary Table 1. Equilibrium climate sensitivity in our 6 CMIP5 models. The CMIP5 ensemble is computed across all CMIP5 models given in Table S2 of Zelinka et al. [1].

	Model	Equilibrium Climate Sensitivity (°C)
CMIP5	ACCESS1.3	3.55
	CSIRO-Mk3-6-0	4.09
	HadGEM2-ES	4.60
	IPSL-CM5A-MR	4.11
	MIROC5	2.72
	NorESM1-M	2.80
	CMIP5 ensemble mean	3.31 ± 0.74

Supplementary Table 2. Equilibrium climate sensitivity in our 5 CMIP6 models. The CMIP5 ensemble is computed across all CMIP5 models given in Table S1 of Zelinka et al. [1].

	Model	Equilibrium Climate Sensitivity (°C)
CMIP6	CESM2	5.15
	CNRM-CM6-1	4.90
	CNRM-ESM2-1	4.79
	MRI-ESM2-0	3.13
	UKESM1-0-LL	5.36
	CMIP6 ensemble mean	3.86 ± 1.10

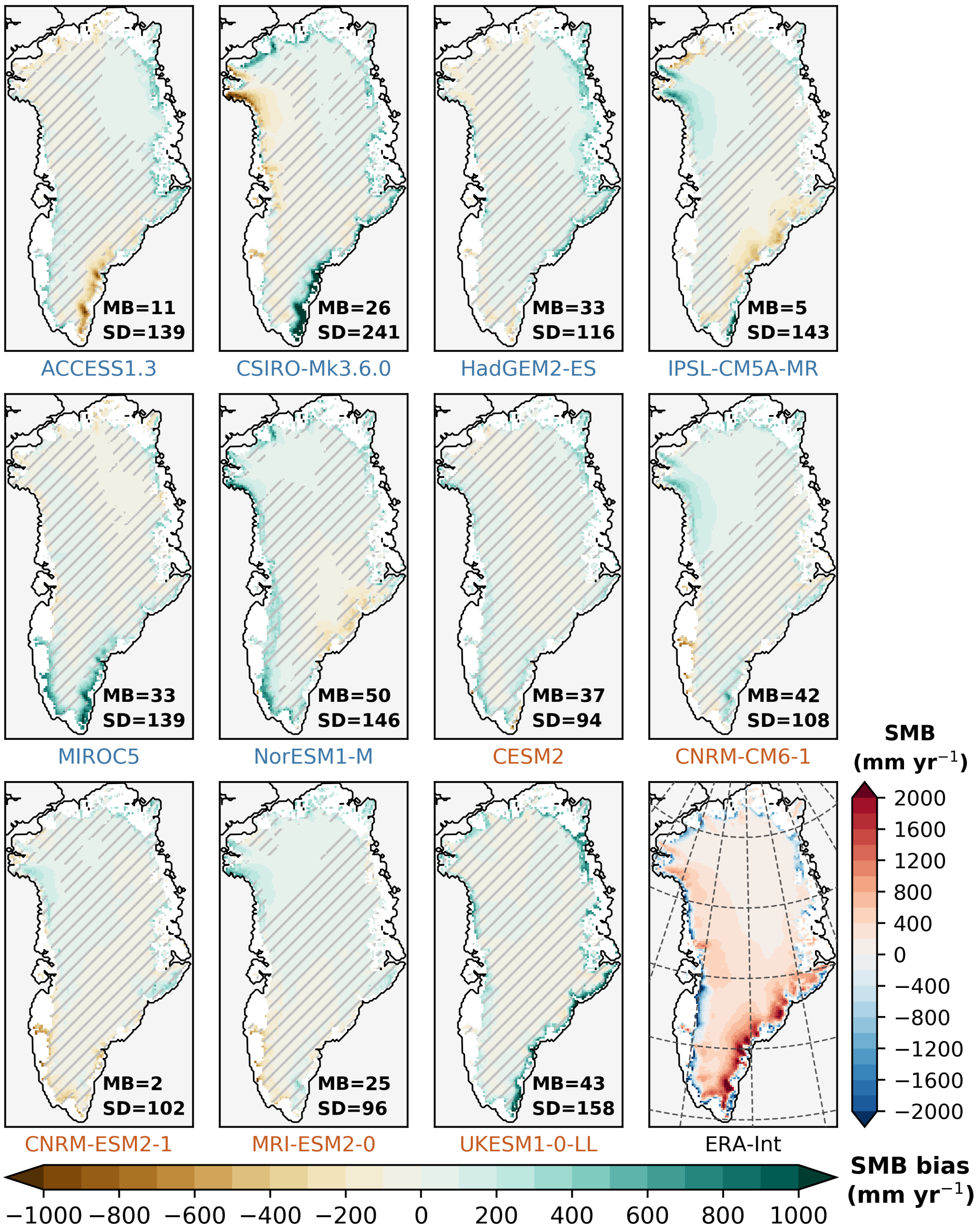

Supplementary Figure 1. **Comparison between the SMB of MAR forced by reanalysis and GCM data over 1981-2010.** Difference (MAR-GCM minus MAR-ERA-Int) of the SMB of MAR forced by the 6 CMIP5 models used in this study (blue labels), 5 CMIP6 models (dark orange) and one reference simulation (bottom right), where MAR was forced by ERA-Interim reanalysis. The presented SMB differences are given in mm water equivalent. "MB" stands for the mean bias and "SD" for the standard deviation. The hatching in the figure indicates pixels where the absolute difference between MAR-GCM minus MAR-ERA-Int is lower than the interannual variability (standard deviation) for a given pixel.

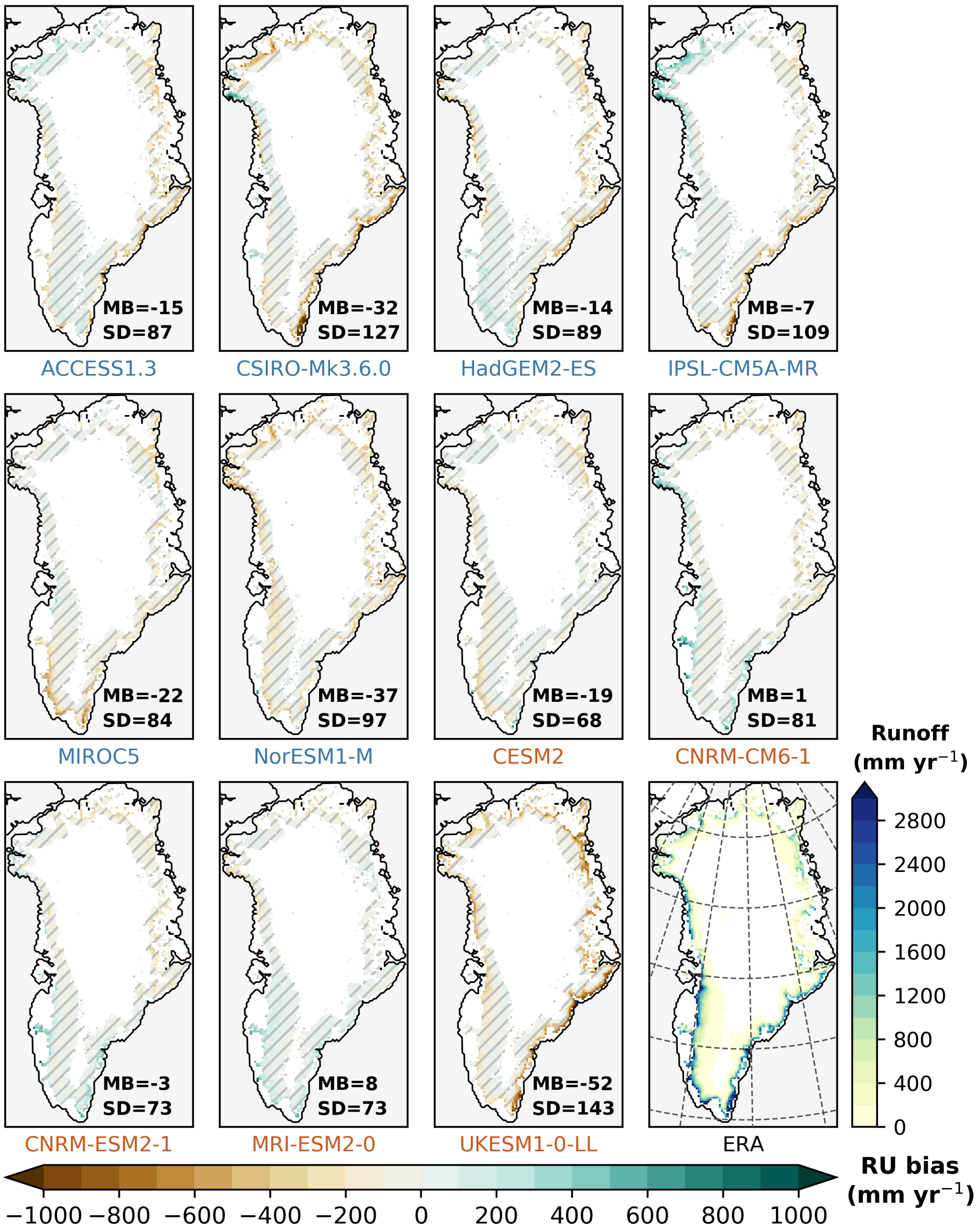

Supplementary Figure 2. **Comparison between the runoff of MAR forced by reanalysis and GCM data over 1981-2010.** Same as Figure S1 but for runoff (mm w.e).

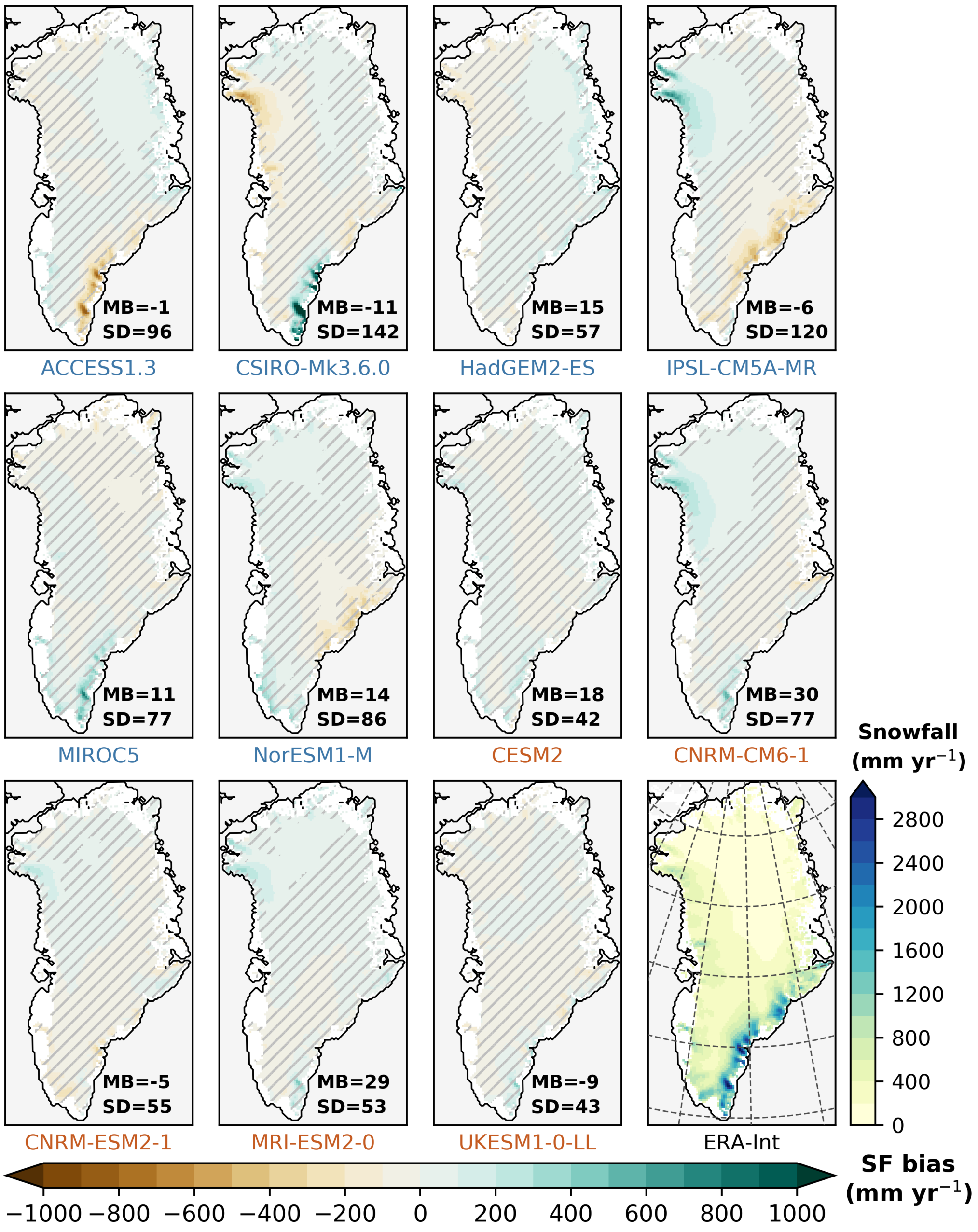

Supplementary Figure 3. **Comparison between the snowfall of MAR forced by reanalysis and GCM data over 1981-2010.** Same as Figure S1 but for snowfall (mm w.e.).

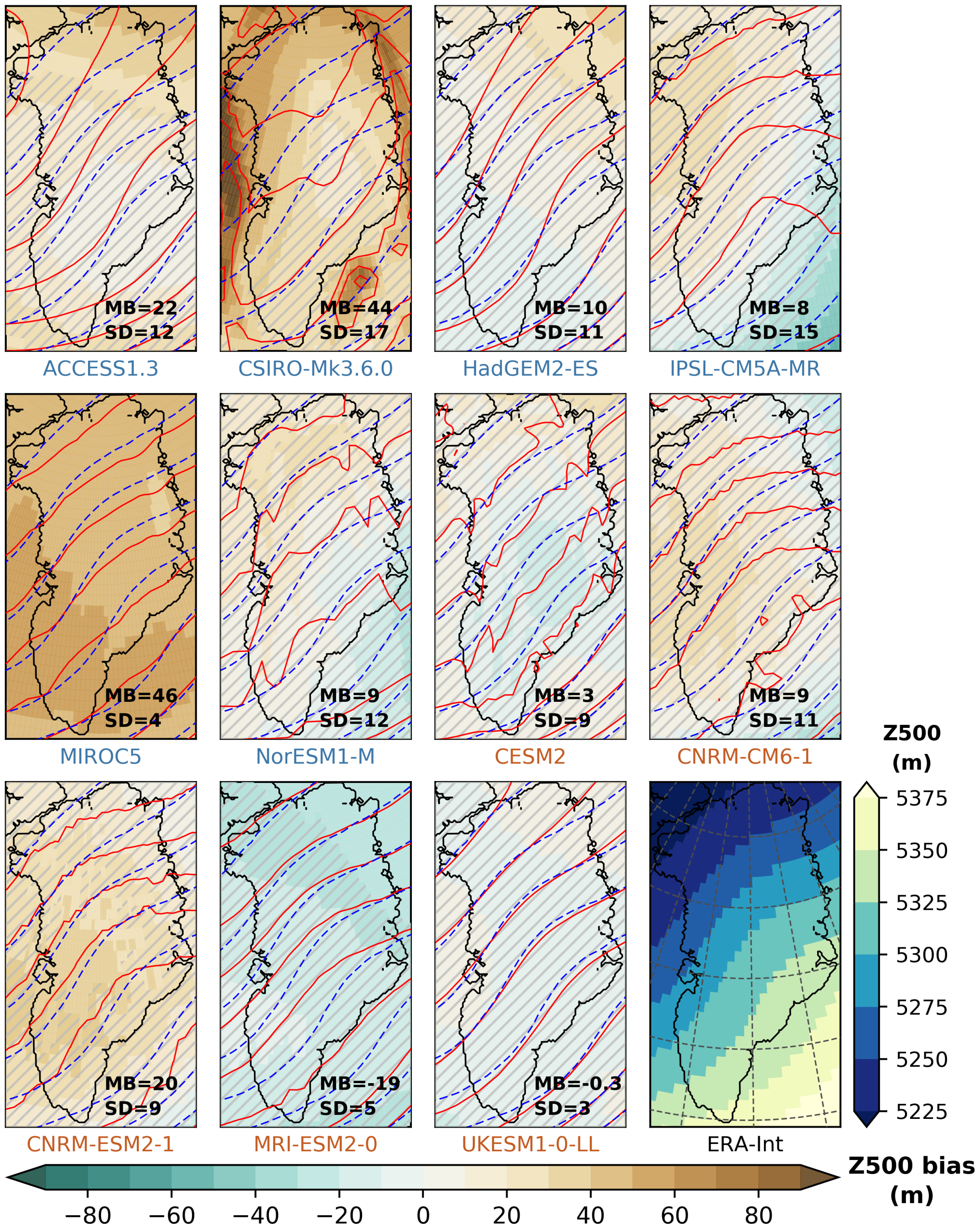

Supplementary Figure 4. **Comparison between the geopotential height at 500 hPa from raw GCM data and ERA-Interim reanalysis during 1981-2010.** Difference (GCM minus ERA-Int) in height of the 500 hPa layer (colors) and the absolute height of the 500 hPa layer, where the red line corresponds to the GCM circulation and the dotted blue lines is the 500 hPa circulation of ERA-Interim.

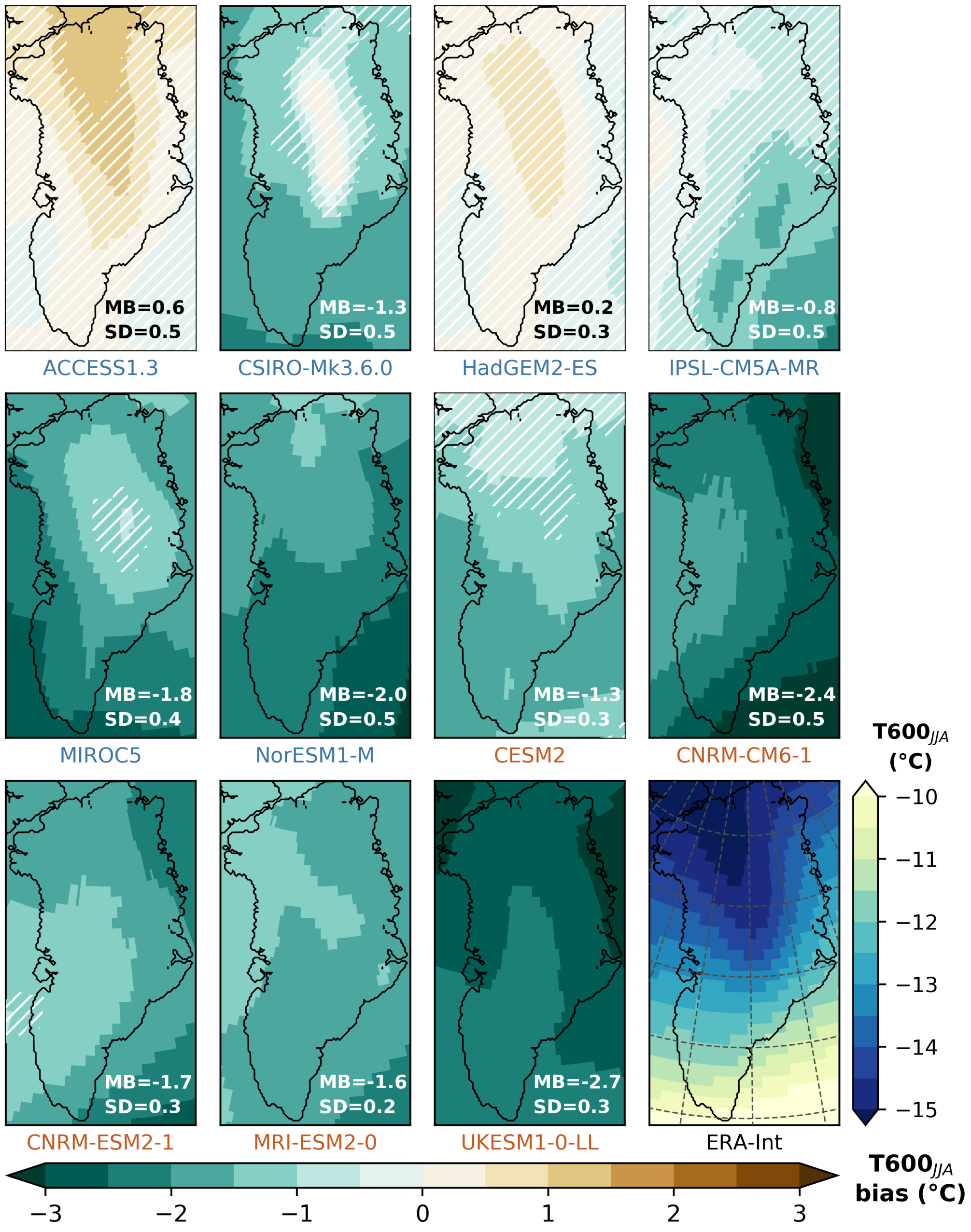

Figure S5. **Comparison between the temperature at 600 hPa from raw GCM data and ERA-Interim reanalysis over 1981-2010.** Same as Figure S4 but for the difference in 600 hPa temperature.

References

[1] Mark D. Zelinka et al. “Causes of Higher Climate Sensitivity in CMIP6 Models”. In: *Geophysical Research Letters* 47.1 (2020), pp. 1–12. ISSN: 19448007. DOI: 10.1029/2019GL085782.
